# Stochastic time‐concentration activity models for cytotoxicity in 3D brain cell cultures

**DOI:** 10.1186/1742-4682-10-19

**Published:** 2013-03-14

**Authors:** Maria Renner, Marie‐Gabrielle Zurich, Annette Kopp‐Schneider

**Affiliations:** 1 Division of Biostatistics, German Cancer Research Center, Im Neuenheimer Feld 280, Heidelberg D‐69120, Germany; 2 Département de Physiologie, Université de Lausanne, 7 rue du Bugnon, Lausanne CH‐1005, Switzerland

**Keywords:** Markov process, Stochastic model, LDH activity, Least squares regression, Maximum likelihood estimation, Likelihood ratio test, Simulation study, Three dimensional brain cell cultures

## Abstract

**Background:**

*In vitro* aggregating brain cell cultures containing all types of brain cells have been shown to be useful for neurotoxicological investigations. The cultures are used for the detection of nervous system‐specific effects of compounds by measuring multiple endpoints, including changes in enzyme activities. Concentration‐dependent neurotoxicity is determined at several time points.

**Methods:**

A Markov model was set up to describe the dynamics of brain cell populations exposed to potentially neurotoxic compounds. Brain cells were assumed to be either in a healthy or stressed state, with only stressed cells being susceptible to cell death. Cells may have switched between these states or died with concentration‐dependent transition rates. Since cell numbers were not directly measurable, intracellular lactate dehydrogenase (LDH) activity was used as a surrogate. Assuming that changes in cell numbers are proportional to changes in intracellular LDH activity, stochastic enzyme activity models were derived. Maximum likelihood and least squares regression techniques were applied for estimation of the transition rates. Likelihood ratio tests were performed to test hypotheses about the transition rates. Simulation studies were used to investigate the performance of the transition rate estimators and to analyze the error rates of the likelihood ratio tests. The stochastic time‐concentration activity model was applied to intracellular LDH activity measurements after 7 and 14 days of continuous exposure to propofol. The model describes transitions from healthy to stressed cells and from stressed cells to death.

**Results:**

The model predicted that propofol would affect stressed cells more than healthy cells. Increasing propofol concentration from 10 to 100 *μ*M reduced the mean waiting time for transition to the stressed state by 50%, from 14 to 7 days, whereas the mean duration to cellular death reduced more dramatically from 2.7 days to 6.5 hours.

**Conclusion:**

The proposed stochastic modeling approach can be used to discriminate between different biological hypotheses regarding the effect of a compound on the transition rates. The effects of different compounds on the transition rate estimates can be quantitatively compared. Data can be extrapolated at late measurement time points to investigate whether costs and time‐consuming long‐term experiments could possibly be eliminated.

## Background

The central nervous system (CNS) is a frequent target for toxic effects of environmental, industrial and pharmaceutical compounds. Natural or artificial toxic compounds can cause irreversible damage to the nervous tissue or even kill the neurons [1]. *In vitro* assays have been developed for the initial identification of potential neurotoxicants. Aggregating brain cell cultures are suited for the detection and study of neurotoxicants for developmental, acute and chronic neurotoxicity [2]‐ [5]. These are three‐dimensional *in vitro* cell cultures prepared from mechanically dissociated embryonic rat brain. The initial cell suspension is kept under continuous gyratory agitation. Spherical aggregates form spontaneously and are maintained in suspension culture for several weeks. Within the aggregates, the cells reconstitute a histotypic brain architecture. The cultures acquire organ‐specific functionality since they contain the different brain cell types (especially neurons, astrocytes and oligodendrocytes) which are able to interact in a physiological manner. High availability, reproducibility and robustness of brain aggregate cell cultures enable routine test procedures for CNS toxicity testing.

**Figure 1:**
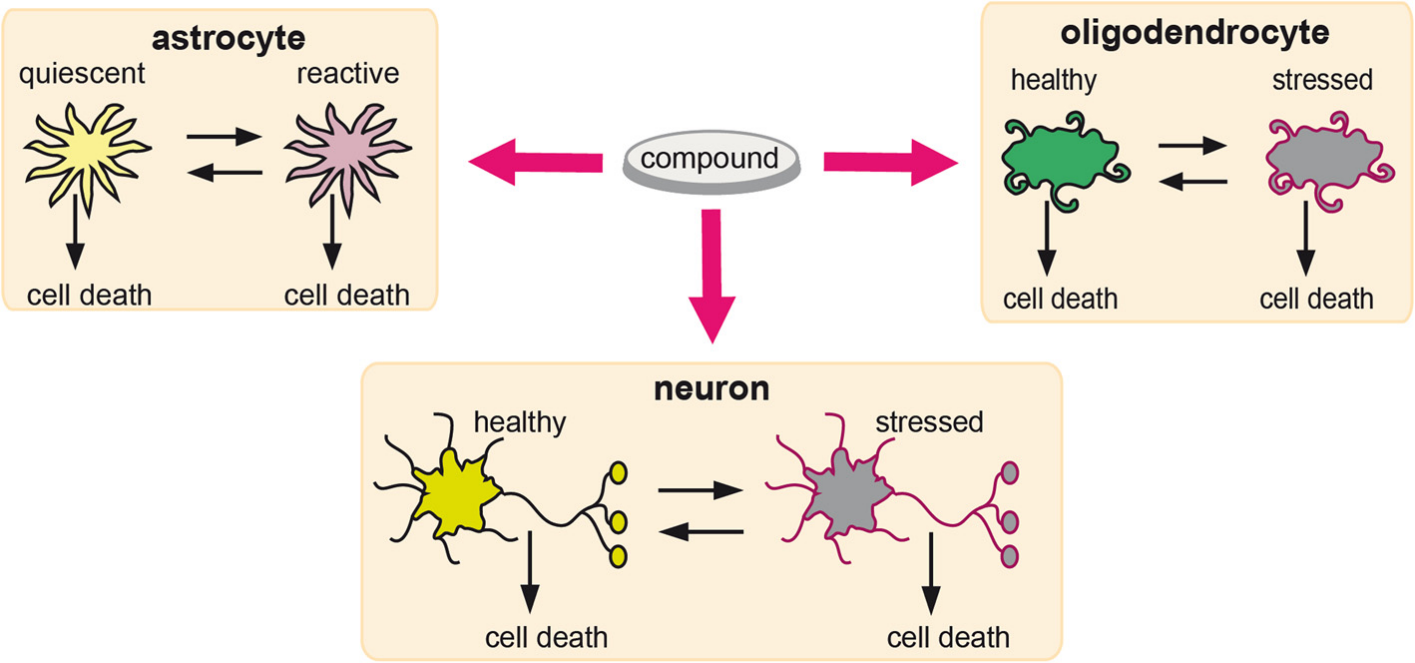
**Compound effects on brain cell populations.** Direct compound effects on the single brain cell populations are modeled. Secondary effects due to interaction between populations are not considered.

Brain aggregate cell cultures are used in studies of chronic toxicity when the cells are highly differentiated. The cultures are exposed to several concentrations of potential neurotoxicants. Besides cell type‐specific enzyme activities, the ubiquitous cytosolic enzyme lactate dehydrogenase (LDH) as indicator for cellular integrity is measured photometrically to assess cell loss. The brain cell types respond differently to injury. Each injured cell type can send signals to the others. The latter will respond to these signals by becoming reactive (astrocytes), stressed (neurons and oligodendrocytes) or by dying. In order to describe the effects of potential neurotoxicants on the behavior and survival of different brain cell types, we set up and applied a mathematical cell population model. For the mathematical modeling approach, the populations of neurons and oligodendrocytes were divided into two subtypes, which we called healthy and stressed. This clearly is a simplification of continual biological processes leading from cellular health to cellular death: nevertheless, a mathematical model represents specific aspects of reality that may be sufficient to answer particular questions (see [6], pp. 4‐6), two of which are investigated in the present study. On the one hand we examined whether the complex mechanisms leading to cellular death of brain cells caused by a toxic compound could be discretized into a stochastic two‐step process: the first step accounts for a period of adaptive and protective mechanisms of cells enhancing cellular survival, whereas the second step describes a critical degree of injury beyond which the cells will not be able to escape cellular death. On the other hand, the effect of the compound on the rates of the process was studied, given that the model correctly reflects the cellular mechanisms. For the modeling approach, the population of astrocytes was also subdivided into two subtypes that we called quiescent and reactive, which can have different susceptibilities to cellular death. Again, a rather complex biological process was simplified so that it could be analyzed by mathematical techniques. Figure 1 illustrates the subtypes of neurons, astrocytes and oligodendrocytes that were modeled.

In the present manuscript, a mathematical model was derived to describe and predict dynamics of brain cell populations that were exposed to a potentially neurotoxic compound. The cell population was modeled as a two‐state Markov process in continuous time with concentration‐dependent transition rates. Since cell numbers are not directly measurable, intracellular LDH activity was used as a surrogate. Intracellular LDH activity is proportional to the living cells, i.e. a cell that contains intracellular LDH is considered to be alive. From the cell population model, stochastic time‐concentration activity models for intracellular LDH activity data were derived, and maximum likelihood and nonlinear least squares regression techniques were used to estimate the transition rates. In computer simulation studies the estimators were compared via their bias, and coverage probabilities for the confidence intervals were determined. Likelihood ratio tests were performed for testing hypotheses about the biologically‐based activity model parameters. The activity model was applied to experimental data, and least squares estimates of the parameters were obtained and interpreted.

**Figure 2:**
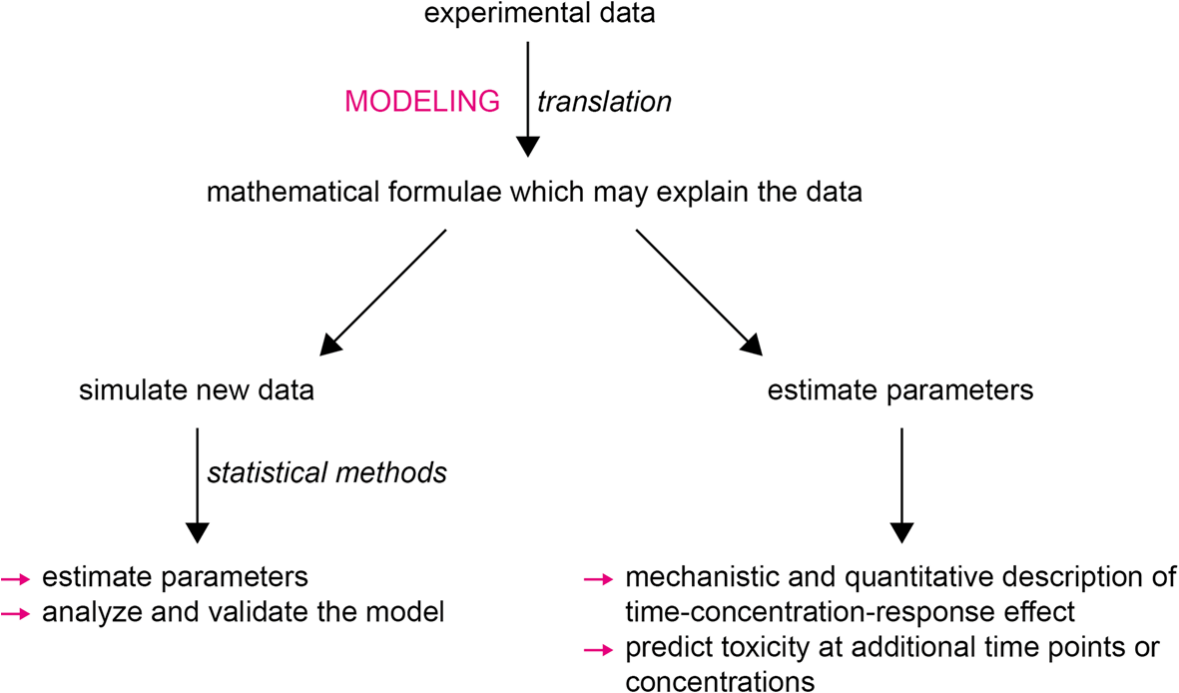
**Procedure of modeling.** Procedure of mathematical modeling in this paper.

The manuscript is organized as illustrated in Figure 2. At first, experimental data are described. The cell population model is set up and the mathematical formulae for the enzyme activity model are derived, which contain biologically‐based parameters. Simulation studies are carried out to assess the performance of parameter estimation. The formulae are applied to experimental data. Parameter estimates can be used to predict toxicity at additional time points or exposure concentrations.

## Experimental data

Aggregating brain cell cultures were grown in flasks containing between 200 ‐ 500 aggregates. Cell cultures were exposed to several concentrations of the compound under study at several time points in independent parallel cultures. As an endpoint for neurotoxicity testing, absolute intracellular LDH activity was monitored (see Koh and Choi [7] pp. 83‐90 for a description of the method). Measurements were independent rather than longitudinal.

**Figure 3:**
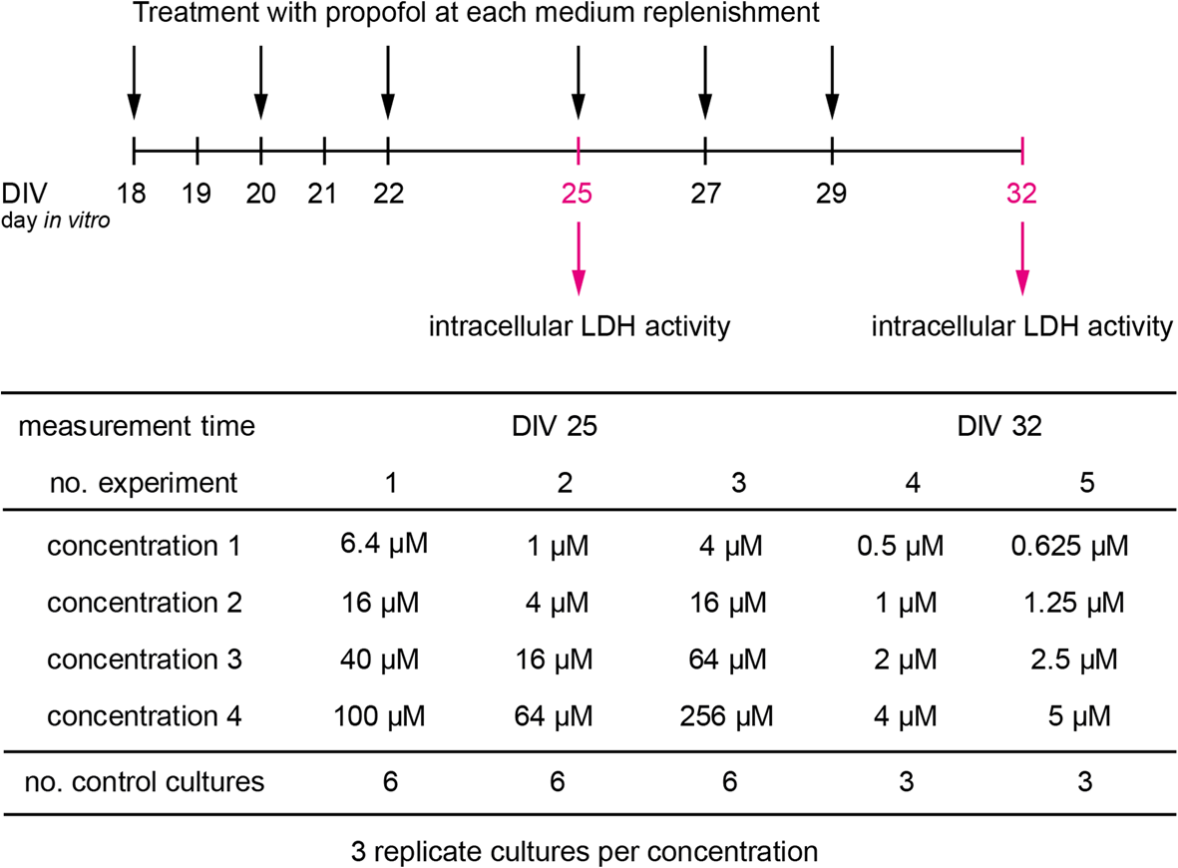
**Design of propofol experiment.** Experimental setup for aggregating brain cell cultures exposed to propofol.

The detailed experimental setup is illustrated in Figure 3. In total, five experiments, in which cultures were harvested and analyzed for intracellular LDH activity, were performed. Measurements were obtained after 7 and 14 days of exposure to propofol. In each experiment, propofol was tested at four concentrations together with a group of solvent cultures. Since analysis of variance with factors *concentration* and *experiment* showed no significant effect of *experiment* at level 5%, measurements from experiments at the same time point were analyzed as if they originated from one experiment at that time point.

## Mathematical model formulation

We model the fate of cells in a brain aggregate. The model parameters are expressed as a function of the concentration of the compound.

### Cell population model

**Figure 4:**
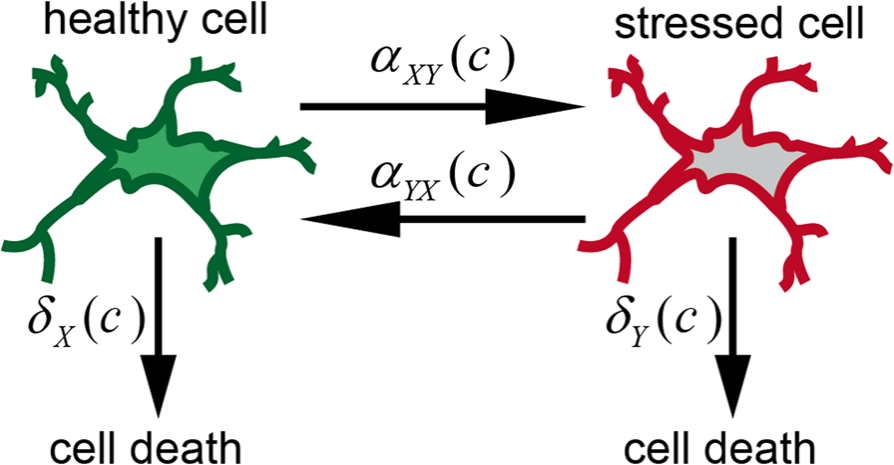
**Cell population model with two states: ‘healthy’ and ‘stressed’.** A brain cell population modeled as a two‐state Markov process in continuous time with transition rates dependent on exposure concentration of a compound.

Cells of a population are assumed to be: a) in one of two states; b) reversibly switch between these two states; or c) die (see Figure 4). Let *X*(*t*,*c*) denote the number of cells in state one and *Y*(*t*,*c*) denote the number of cells in state two at time *t* and concentration *c*. In a small time interval [*t*,*t*+*Δt*) the following events can occur: 

•a cell in state one can become a cell in state two with probability *α*_*XY*_(*t*,*c*)*Δt* + *o*(*Δt*),

•a cell in state two can become a cell in state one with probability *α*_*YX*_(*t*,*c*)*Δt* + *o*(*Δt*),

•a cell in state one can die with probability *δ*_*X*_(*t*,*c*)*Δt* + *o*(*Δt*),

•a cell in state two can die with probability *δ*_*Y*_(*t*,*c*)*Δt* + *o*(*Δt*),

•more than one event at the same time instant occurs with probability *o*(*Δt*).

Assume the system starts at time *t*=0 with *X*(0,*c*)=*x*_0_ and *Y*(0,*c*)=*y*_0_ for all *c*≥0 and that cells act independently of each other. With these definitions, the process (*X*(*t*,*c*),*Y*(*t*,*c*)) is a Markov process in continuous time. Let $P_{i,j}(t,c):=P[X(t,c)=i,Y(t,c)=j]$ denote the joint distribution of (*X*(*t*,*c*),*Y*(*t*,*c*)). Every cell will be in one of three states, namely healthy, stressed or dead. Hence the process (*X*(*t*,*c*),*Y*(*t*,*c*),*x*_0_+*y*_0_−*X*(*t*,*c*)−*Y*(*t*,*c*)) follows a multinomial distribution. The distribution is derived analytically via the generating function denoted by

(1) $$\begin{array}{lll} \Psi_{x_{0},y_{0}}(x,y,t,c) & =E\left[x^{X(t,c)}y^{Y(t,c)}|X(0,c)=x_{0},Y(0,c)=y_{0}\right]=\underset{i,j}{\sum }x^{i}y^{j}P_{i,j}(t,c)\; & \;\\ & =\Psi_{1,0}{\left(x,y,t,c\right)}^{x_{0}}\cdot \Psi_{0,1}{\left(x,y,t,c\right)}^{y_{0}}\; \end{array}$$

for a process (*X*(*t*,*c*),*Y*(*t*,*c*)) which starts in state (*x*_0_,*y*_0_) at time *t*=0 for any concentration *c*≥0. The last equality in (1) holds due to the independence of the cells since a process starting with *x*_0_ cells in state one and *y*_0_ cells in state two can be viewed as a sum of *x*_0_ processes starting with one cell in state one and *y*_0_ processes starting with one cell in state two.

Assume *X*(0,*c*)=*x*_0_≥1 and *Y*(0,*c*)=0, i.e. that all cells start in state one at time *t*=0 for all *c*≥0. Due to (1) it is sufficient to solve the Kolmogorov forward equations (see e.g. Feller [8]) for *x*_0_=1,*y*_0_=0. These are given by **P’**=*A***P**, i.e.

$$\left(\begin{array}{c} P_{1,0}^{\prime }(t,c)\\ P_{0,1}^{\prime }(t,c) \end{array}\right)=\left(\begin{array}{cc} -\left(\delta_{X}(t,c)+\alpha_{\text{XY}}(t,c)\right) & \alpha_{\text{YX}}(t,c)\\ \alpha_{\text{XY}}(t,c) & -\left(\delta_{Y}(t,c)+\alpha_{\text{YX}}(t,c)\right) \end{array}\right)\cdot \left(\begin{array}{c} P_{1,0}(t,c)\\ P_{0,1}(t,c) \end{array}\right),$$

with initial condition

$${\text{P}}_{0}=\left(\begin{array}{c} P_{1,0}(0,c)\\ P_{0,1}(0,c) \end{array}\right)=\left(\begin{array}{c} 1\\ 0 \end{array}\right).$$

The Kolmogorov forward equations are solved analytically for time‐independent transition rates by a matrix exponential, i.e. **P**=*e*^*λt*^**vP**_0_ where **v** is the eigenvector of *A* to the eigenvalue *λ*, i.e. *A***v**=*λ***v**. Explicitly the solutions are given by

$$\begin{array}{lll} P_{1,0}(t,c) & =\frac{[\lambda_{1}(c)+\delta_{Y}(c)+\alpha_{\text{YX}}(c)]\cdot e^{\lambda_{1}(c)t}-[\lambda_{2}(c)+\delta_{Y}(c)+\alpha_{\text{YX}}(c)]\cdot e^{\lambda_{2}(c)t}}{\lambda_{1}(c)-\lambda_{2}(c)}\; & \;\\ P_{0,1}(t,c) & =\alpha_{\text{XY}}(c)\cdot \frac{e^{\lambda_{1}(c)t}-e^{\lambda_{2}(c)t}}{\lambda_{1}(c)-\lambda_{2}(c)}\; & \; \end{array}$$

where *λ*_1_(*c*)≠*λ*_2_(*c*) are the real solutions of the characteristic equation

$$\begin{array}{c} \lambda^{2}(c)+[\delta_{X}(c)+\alpha_{\text{XY}}(c)+\delta_{Y}(c)+\alpha_{\text{YX}}(c)]\cdot \lambda (c)+\alpha_{\text{YX}}(c)\delta_{X}(c)+\delta_{Y}(c)\delta_{X}(c)+\alpha_{\text{XY}}(c)\delta_{Y}(c)=0. \end{array}$$

The solutions are continuously extended for the limiting case *λ*_1_(*c*)=*λ*_2_(*c*), which holds if and only if

$$\alpha_{\text{YX}}(c)=0\;\text{and}\;\delta_{X}(c)=\delta_{Y}(c)-\alpha_{\text{XY}}(c)$$

or

$$\alpha_{\text{XY}}(c)=0\;\text{and}\;\delta_{X}(c)=\delta_{Y}(c)+\alpha_{\text{YX}}(c).$$

The solution for *P*_0,0_(*t*,*c*) is given by

$$P_{0,0}(t,c)=1-P_{1,0}(t,c)-P_{0,1}(t,c).$$

The joint distribution of (*X*(*t*,*c*),*Y*(*t*,*c*)) is given by

Ψx0,0(x,y,t,c)=Ψ1,0(x,y,t,c)x0⇔∑i,jxiyjPi,j(t,c)=P0,0(t,c)+xP1,0(t,c)+yP0,1(t,c)x0⇔∑k=0x0∑l=0kxlyk−lPl,k−l(t,c)=∑k=0x0∑l=0kx0kklP0,0x0−k(t,c)·xP1,0(t,c)l·yP0,1(t,c)k−l.

As expected the distribution of (*X*(*t*,*c*),*Y*(*t*,*c*)) follows a multinomial distribution

(2) $$P_{l,k-l}(t,c)=\frac{x_{0}!}{l!\;(k-l)!\;(x_{0}-k)!}\cdot P_{1,0}^{l}(t,c)\cdot P_{0,1}^{k-l}(t,c)\cdot P_{0,0}^{x_{0}-k}(t,c),$$

for all *k*=0,...,*x*_0_ and *l*=0,...,*k* with $P_{x_{0},0}(0,c)=1$ and *P*_*i*,*j*_(0,*c*)=0 for all (*i*,*j*)≠(*x*_0_,0) and *c*≥0.

The expected values of (*X*(*t*,*c*),*Y*(*t*,*c*)) are given by

$$\begin{array}{lll} E[X(t,c)] & =x_{0}\cdot P_{1,0}(t,c),\; & \;\\ E[Y(t,c)] & =x_{0}\cdot P_{0,1}(t,c).\; & \; \end{array}$$

Solutions of the Kolmogorov forward equations and the joint distribution of (*X*(*t*,*c*),*Y*(*t*,*c*)) can analogously be derived for *x*_0_=0 and *y*_0_≥1.

### Activity models

Cell numbers cannot directly be observed experimentally and therefore enzyme activity data are used as a surrogate for the cell number. Absolute enzyme activity measurements strongly fluctuate between experiments. For comparability between experiments absolute enzyme activity measurements are normalized. Depending on the aim of the experimental study, two normalization approaches can be taken. In the first approach, time activity data are normalized with respect to time point zero to determine effects of time on untreated cultures. For concentration *c*=0 absolute enzyme activities at time *t* are divided by the mean of absolute enzyme activities at time *t*=0. A time activity model is derived for this case. In the second approach, time‐concentration activity data are normalized with respect to an untreated control group at the same time point. Absolute enzyme activities at time *t* and concentration *c* are divided by the mean of absolute enzyme activities at time *t* and concentration *c*=0. A time‐concentration activity model is derived for this case. Note that in general the time activity model is not a special case of the time‐concentration activity model since data differ with respect to the normalization procedure.

#### Time activity model

In this model, transition rates are constant and the dependence of cell numbers, enzyme activity and probability functions on concentration *c* is omitted for notational convenience. In the absence of exposure the time course of enzyme activity of untreated cells is modeled by

(3) $$A(t)=\frac{X(t)+Y(t)}{X(0)+Y(0)}=\frac{X(t)+Y(t)}{x_{0}}.$$

The following assumptions are made: 

A1 The number of cells at time zero is given by: *X*(0)=*x*_0_, *Y*(0)=0.

A2 Cells in state one cannot die: *δ*_*X*_=0.

A3 The transition from state one to state two occurs with positive probability: *α*_*XY*_>0.

A4 Cells in state two can die with positive probability: *δ*_*Y*_>0.

The sum *X*(*t*)+*Y*(*t*) follows a binomial distribution: $X(t)+Y(t)∼B(x_{0},1-P_{0,0}(t))$. The solutions of the characteristic equation depend only on the sum of the transition rates and the product *δ*_*Y*_·*α*_*XY*_: *λ*_1/2_=−*p*±*w*, where *p*=(*δ*_*Y*_+*α*_*XY*_+*α*_*YX*_)/2∈(0,*∞*) and $w=\sqrt{p^{2}-\delta_{Y}\alpha_{\text{XY}}}\in [0,p)$. Hence not all three transition rates are identifiable via *P*_0,0_(*t*), and the parameterization of *P*_0,0_(*t*) only depends on *p* and *w*:

$$\begin{array}{c} P_{0,0}(t)=\left\{\begin{array}{cc} 1-\left[(p+w)\cdot e^{(-p+w)t}-(p-w)\cdot e^{-(p+w)t}\right]/(2w) & ,w>0\;(\delta_{Y}\neq \alpha_{\text{XY}}\;\text{or}\;\alpha_{\text{YX}}>0)\\ 1-e^{-\delta_{Y}t}\cdot (1+\delta_{Y}t) & ,p=\delta_{Y}=\alpha_{\text{XY}},w=\alpha_{\text{YX}}=0 \end{array}\right). \end{array}$$

#### Time‐concentration activity model

In this model, transition rates are assumed to be linear functions of concentration. Enzyme activity of cells exposed to several concentrations *c* of a compound at time *t* is modeled by

(4) $$A(t,c)=\frac{X(t,c)+Y(t,c)}{\text{mean}[X(t,0)+Y(t,0)]},$$

where the denominator denotes the arithmetic mean of replicate measurements of untreated control *c*_1_,...,*c*_*r*_=0, i.e. $\text{mean}[X(t,0)+Y(t,0)]=\sum_{l=1}^{r}(X(t,c_{l})+Y(t,c_{l}))/r$. The transition rates are defined as linear functions of concentration, i.e. *δ*_*X*_(*c*)=*δ*_*X*__*i*_+*c*·*δ*_*X*__*s*_,*δ*_*Y*_(*c*)=*δ*_*Y*__*i*_+*c*·*δ*_*Y*__*s*_,*α*_*XY*_(*c*)=*α*_*XY*__*i*_+*c*·*α*_*XY*__*s*_ and *α*_*YX*_(*c*)=*α*_*YX*__*i*_+*c*·*α*_*YX*__*s*_. The subscript *i* denotes *intercept* parameters, i.e. parameters that are independent of concentration, whereas the subscript *s* denotes *slope* parameters, i.e. parameters that account for the linear effect of concentration. In addition to A1 the following assumptions are made: 

A2* Cells in state one cannot die: *δ*_*X*__*i*_=*δ*_*X*__*s*_=0.

A3* Cells in state two cannot switch back to state one: *α*_*YX*__*i*_=*α*_*YX*__*s*_=0.

Since the compounds under study are potentially toxic, these assumptions reflect that the probability of a stressed cell becoming healthy or a healthy cell dying are negligible compared to probability of a healthy cell becoming stressed or a stressed cell dying. Note that if *δ*_*Y*__*i*_=0 or *α*_*XY*__*i*_=0, cells from the control culture cannot die. In this case *X*(*t*,0)+*Y*(*t*,0)≡*x*_0_ for all *t*≥0 and hence *A*(*t*,*c*) is a generalization of *A*(*t*).

To calculate the distribution of *A*(*t*,*c*) we approximated the binomial distribution of *X*(*t*,*c*)+*Y*(*t*,*c*) by a normal distribution. Let Var denote the variance. If $0<\text{Var}\left(\text{mean}[X(t,0)+Y(t,0)]\right)\ll E[X(t,0)+Y(t,0)]$, i.e. P[X(t,0)+Y(t,0)>0]→1, then according to Hinkley [9], *A*(*t*,*c*) approximately follows a normal distribution: $A(t,c)∼N(\mu_{A}(t,c),\sigma_{A}^{2}(t,c))$ with

$$\begin{array}{lll} \mu_{A}(t,c) & =\frac{E[X(t,c)+Y(t,c)]}{E[X(t,0)+Y(t,0)]}=\frac{1-P_{0,0}(t,c)}{1-P_{0,0}(t,0)},\; & \;\\ \sigma_{A}^{2}(t,c) & =\frac{\text{Var}[X(t,c)+Y(t,c)]}{{\left(E[X(t,0)+Y(t,0)]\right)}^{2}}=\frac{\left(1-P_{0,0}(t,c))\cdot P_{0,0}(t,c\right)}{x_{0}\cdot {\left(1-P_{0,0}(t,0)\right)}^{2}}.\; & \; \end{array}$$

Note that it must be verified whether $0<\text{Var}\left(\text{mean}[X(t,0)+Y(t,0)]\right)\ll E[X(t,0)+Y(t,0)]$ holds for the parameters at all times *t*≥0.

Figure 4 and the parameter restrictions A2* and A3* imply that *A*(*t*,*c*) is symmetric in *δ*_*Y*_(*c*)=*δ*_*Y*__*i*_+*c*·*δ*_*Y*__*s*_ and *α*_*XY*_(*c*)=*α*_*XY*__*i*_+*c*·*α*_*XY*__*s*_:

$$\begin{array}{c} P_{0,0}(t,c)=\left\{\begin{array}{cc} 1-\left[\alpha_{\text{XY}}(c)\cdot e^{-\delta_{Y}(c)\cdot t}-\delta_{Y}(c)\cdot e^{-\alpha_{\text{XY}}(c)\cdot t}\right]/(\alpha_{\text{XY}}(c)-\delta_{Y}(c)) & ,\delta_{Y}(c)\neq \alpha_{\text{XY}}(c)\\ \;1-e^{-\delta_{Y}(c)\cdot t}\cdot (1+\delta_{Y}(c)\cdot t) & ,\delta_{Y}(c)=\alpha_{\text{XY}}(c) \end{array}\right). \end{array}$$

Exchangeability of the pairs of parameters (*δ*_*Y*__*i*_,*δ*_*Y*__*s*_) and (*α*_*XY*__*i*_,*α*_*XY*__*s*_) is removed by requiring *δ*_*Y*__*i*_≤*α*_*XY*__*i*_ in order to prevent optimization problems.

## Parameter estimation for activity models

Let (*a*_*i*_,*t*_*i*_,*c*_*i*_) for *i*=1,...,*n* denote *n* measurements of activities at time points and concentrations. Model parameters are estimated preferably by maximum likelihood techniques. We perform least squares regression as an alternative approach to parameter estimation since it is easily implemented.

### Maximum likelihood estimation

Parameters from the time activity model can be estimated by minimization of the negative log‐likelihood

$$-\log L=-\overset{n}{\underset{i=1}{\sum }}x_{0}a_{i}\log(1-P_{0,0}(t_{i}))+x_{0}(1-a_{i})\log P_{0,0}(t_{i}).$$

Parameters from the time‐concentration activity model can be estimated by minimizing the approximate negative log‐likelihood

$$-\log L=\overset{n}{\underset{i=1}{\sum }}\frac{1}{2}\cdot \frac{{\left(a_{i}-\mu_{A}(t_{i},c_{i})\right)}^{2}}{\overset{2}{\underset{A}{\sigma }}(t_{i},c_{i})}+\log\sigma_{A}(t_{i},c_{i}).$$

As noted before, it has to be verified whether the estimated parameter combinations obtained from experimental data lead to $0<\text{Var}\left(\text{mean}[X(t,0)+Y(t,0)]\right)\ll E[X(t,0)+Y(t,0)]$ for all times *t*≥0. In case this condition is not fulfilled, maximum likelihood estimation cannot be relied on.

### Least squares estimation

For the time activity model, $E[A(t)]=E[X(t)+Y(t)]/x_{0}$ according to (3). For least squares regression we assume that

$$a(t_{i})=E[A(t_{i})]+\varepsilon_{i}=1-P_{0,0}(t_{i})+\varepsilon_{i},$$

where $\varepsilon_{1},\ldots ,\varepsilon_{n}∼N(0,\sigma^{2})$ are independent measurement errors. The least squares estimator minimizes

$$\text{RSS}=\overset{n}{\underset{i=1}{\sum }}{\left[a_{i}-(1-P_{0,0}(t_{i}))\right]}^{2}.$$

For the time‐concentration activity model $E[A(t,c)]=E\left[\frac{X(t,c)+Y(t,c)}{\text{mean}[X(t,0)+Y(t,0)]}\right]$ according to (4). The expected value can be approximated by a second order Taylor series expansion, i.e.

$$\begin{array}{c} E[A(t,c)]\approx \frac{E[X(t,c)+Y(t,c)]}{E[X(t,0)+Y(t,0)]}+\text{Var}\left(\text{mean}[X(t,0)+Y(t,0)]\right)\cdot \frac{E[X(t,c)+Y(t,c)]}{{\left(E[X(t,0)+Y(t,0)]\right)}^{3}}. \end{array}$$

Note that this approximation of the expected value of *A*(*t*,*c*) differs from *μ*_*A*_(*t*,*c*). Analogously we assume that

$$a(t_{i},c_{i})=E[A(t_{i},c_{i})]+\varepsilon_{i}=\frac{1-P_{0,0}(t_{i},c_{i})}{1-P_{0,0}(t_{i},0)}+\frac{\left(1-P_{0,0}(t_{i},c_{i}))\cdot P_{0,0}(t_{i},0\right)}{r\cdot x_{0}\cdot {\left(1-P_{0,0}(t_{i},0)\right)}^{2}}+\varepsilon_{i},$$

where *r* denotes the number of replicate measurements of the control group. The least squares estimator minimizes

$$\text{RSS}=\overset{n}{\underset{i=1}{\sum }}{\left[a_{i}-\frac{1-P_{0,0}(t_{i},c_{i})}{1-P_{0,0}(t_{i},0)}-\frac{\left(1-P_{0,0}(t_{i},c_{i})\right)\cdot P_{0,0}(t_{i},0)}{r\cdot x_{0}\cdot {\left(1-P_{0,0}(t_{i},0)\right)}^{2}}\right]}^{2}.$$

### Performance of estimation

Let $\theta ={\left(\theta_{k}\right)}_{k=1,\mathrm{...},m}\in R^{m}$ denote the parameter to be estimated and ${\hat{\theta }}_{i}\in R^{m}$, *i*=1,...,*N* be *N* independent estimates of *θ*. The relative bias in each component *θ*_*k*_,*k*=1,...,*m* of *θ* is given by $B(\theta_{k})=N^{-1}\sum_{i=1}^{N}({\hat{\theta }}_{i_{k}}-\theta_{k})/\theta_{k}$. Wald confidence intervals with a 95% confidence level are calculated from the Jacobian from the optimization algorithm for least squares estimation and from the Hessian from the optimization algorithm for maximum likelihood estimation. Coverage probabilities for confidence intervals are determined to investigate the actual probability that the confidence interval contains the true parameter.

## Testing hypotheses about transition rates

If there is biological evidence that a subset of parameters are identical, e.g. *δ*_*Y*__*i*_=*α*_*XY*__*i*_, a likelihood ratio (LR) test can be performed to compare nested model fits (see Fahrmeir and Hamerle [10], pp. 76 ‐ 78). Under the null hypothesis *H*_0_ that the parameter vector $\theta \in R^{m}$ can be parameterized in the simpler form *θ*=*g*(*θ*_0_) with rank rk(*∂g*(*θ*_0_)/*∂θ*_0_)=*k*<*m*, the test statistic $2\cdot \left(\log L(\hat{\theta })-\log L(\hat{\theta_{0}})\right)$ calculated with maximum likelihood estimates for *θ* and *θ*_0_ is asymptotically $\chi_{\text{df}}^{2}$ distributed with *df*=*m*−*k* degrees of freedom. *H*_0_ can be rejected at significance level *α*, if $2\cdot \left(\log L(\hat{\theta })-\log L(\hat{\theta_{0}})\right)>\chi_{\text{df}}^{2}(1-\alpha )$, where $\chi_{\text{df}}^{2}(1-\alpha )$ is the 1 ‐ *α* quantile of the $\chi_{\text{df}}^{2}$ distribution. If there is biological evidence that a parameter is zero, e.g. *δ*_*Y*__*s*_=0, the distribution of the test statistic under the null hypothesis is a mixture of chi‐squared distributions (see Self and Liang [11]). If maximum likelihood estimation cannot be relied on, an extra sum of squares analysis for nested models as described in Bates and Watts, pp.103 ‐ 104 [12] can be performed.

## Model simulation

For both models the process (*X*(*t*,*c*),*Y*(*t*,*c*)) is simulated according to its multinomial distribution (2) at predefined time points and concentrations, i.e. snapshots of the Markov process are taken. Motivated by the cell number in the brain aggregate culture system, we assume that the system starts with *x*_0_=10^6^. Activities are simulated for one time unit at ten equidistant time points in three replicates.

A different dynamic range consisting of ‘Fast’, ‘Normal’ or ‘Slow’ with ‘high stress’ and ‘low stress’ scenarios, i.e. scenarios with a very small or a very high transition rate *α*_*YX*_, is chosen (see Table 1) such that expected activities lie between 0% ‐ 90%.

**Table 1:** Transition rates for simulation studies of time activity and time‐concentration activity models

		***A*( *t *)**		***A*( *t *,*c)***			
**Scenario**	***δ***_***Y***_	***α***_***XY***_	***α***_***YX***_	$\delta_{Y_{i}}$	$\alpha_{XY_{i}}$	$\delta_{Y_{s}}$	$\alpha_{XY_{s}}$
‘Fast low stress’	10	10	30	‐	‐	‐	‐
‘Fast high stress’	35	15	0	‐	‐	‐	‐
‘Normal low stress’	3.5	2	4.5	‐	‐	‐	‐
‘Normal high stress’	6	4	0	‐	‐	‐	‐
‘Slow low stress’	0.5	0.5	0.5	‐	‐	‐	‐
‘Slow high stress’	1.0	0.5	0	‐	‐	‐	‐
‘Different parameters’	‐	‐	‐	1.1	3.3	1.7	5.1
‘Identical intercept’	‐	‐	‐	2.2	2.2	5.1	1.7
‘Identical slope’	‐	‐	‐	1.1	3.3	8.4	8.4
’Identical rates’	‐	‐	‐	2.2	2.2	3.4	3.4

Transition rates are denoted by *δ*_*Y*_(*c*)≡*δ*_*Y*_, *α*_*XY*_(*c*)≡*α*_*XY*_, *α*_*YX*_(*c*)≡*α*_*YX*_ in [time] ^−1^ for the time activity model and by *δ*_*Y*_(*c*)=*δ*_*Y*__*i*_+*c*·*δ*_*Y*__*s*_, *α*_*XY*_(*c*)=*α*_*XY*__*i*_+*c*·*α*_*XY*__*s*_ for the time‐concentration activity model in [time] ^−1^ and [time · concentration] ^−1^ for intercept and slope parameters, respectively.

Data from the time‐concentration activity model are simulated at five exposure concentrations and control. We choose representative parameter combinations for which $0<\text{Var}\left(\text{mean}[X(t,0)+Y(t,0)]\right)\ll E[X(t,0)+Y(t,0)]$ holds for all 0≤*t*≤1 for a detailed simulation study (see Table 1).

## Implementation

The MATLAB environment is used to simulate dynamics of cell numbers by the function *mnrnd*. Log‐likelihood functions as well as maximum likelihood and least squares parameter estimation are implemented by the functions *fmincon* and *lsqcurvefit* respectively.

## Application to simulated data

### Time activity model

#### Performance of estimation procedure

Table 2 shows the relative bias of least squares and maximum likelihood estimates as well as coverage probabilities with 95% confidence intervals for the time activity model *A*(*t*). A coverage probability of 90.25% was expected for the joint confidence intervals of *p* and *w*, which is the product of the single 95% confidence levels. Maximum likelihood and least squares confidence intervals had coverage probabilities between 90% ‐ 97% and 61% ‐ 97%, respectively. Least squares and maximum likelihood estimators were nearly unbiased for all scenarios.

**Table 2:** Performance of parameter estimation for the time activity model

	**B( *p *) ×10**^**−3**^		**B( *w *) ×10**^**−3**^		**Coverage probability**	
**Model*A(t)***	**LS**	**ML**	**LS**	**ML**	**LS**	**ML**
‘Fast low stress’	.057	.053	.062	.057	.97 [.96,.98]	.97 [.96,.98]
‘Fast high stress’	.032	.029	.076	.069	.61 [.58,.64]	.97 [.95,.97]
‘Normal low stress’	.009	−.014	.001	−.017	.96 [.95,.97]	.96 [.94,.97]
‘Normal high stress’	−.013	−.016	−26.8	−29.7	.95 [.93,.96]	.96 [.95,.97]
‘Slow low stress’	3.2	−.138	5.4	‐.303	.91 [.89,.93]	.94 [.92,.95]
‘Slow high stress’	.090	.006	4.0	−1.7	.92 [.90,.93]	.90 [.88,.92]

Bias **B** of least squares (LS) and maximum likelihood (ML) estimators and coverage probability with a 95% confidence interval of joint estimates for the time activity model determined from 1000 simulations and fits of the scenarios each.

#### Testing hypotheses about transition rates

The limiting case *p*=*δ*_*Y*_=*α*_*XY*_, *w*=*α*_*YX*_=0 was simulated 1000 times for three different dynamic ranges: ‘fast’ (*p*=10), ‘normal’ (*p*=1.5) or ‘slow’ (*p*=.5). LR tests of *H*_0_*:w*=0 versus *H*_1_:*w*>0 were performed at the significance level of 5% to investigate whether a restricted model could be discriminated from the full model. According to Self and Liang [11] the test statistic under *H*_0_ is distributed according to a 50:50 mixture of $\chi_{0}^{2}$** and**$\chi_{1}^{2}$ distributions. Simulating ‘slow’ resulted in 11 cases in which the full model showed a significantly better fit than the restricted model. Simulating ‘normal’ resulted in 36 cases in which the full model showed a significantly better fit than the restricted model. Simulating ‘fast’ resulted in 20 cases in which the full model showed a significantly better fit than the restricted model. Hence the observed type I error of the LR tests was between 1.1% ‐ 3.6%. All 1000 simulations of the other scenarios for *A*(*t*) given in Table 1 led to the rejection of a restricted parameterization, showing that the LR test achieved a power of 100%.

### Time‐concentration activity model

#### Performance of estimation procedure

Table 3 shows the relative bias of least squares and maximum likelihood estimates as well as coverage probabilities with 95% confidence intervals for the time‐concentration activity model *A*(*t*,*c*). A coverage probability of 81.75% was expected for the joint confidence intervals of *δ*_*Y*__*i*_,*α*_*XY*__*i*_,*δ*_*Y*__*s*_ and *α*_*XY*__*s*_, which is the product of the single confidence levels. The coverage probabilities for maximum likelihood and least squares confidence intervals were between 58% ‐ 78% and 77% ‐ 87%, respectively. Confidence intervals of maximum likelihood estimates were smaller than least squares confidence intervals (not shown), resulting in lower coverage probabilities for maximum likelihood confidence intervals. Accuracy of estimation could be improved when the number of replicates was increased. For example, by increasing the number of replicates from 3 to 10 for the scenario ‘Identical slope,’ the coverage probabilities for maximum likelihood and least squares confidence intervals increased from 77% to 89% and from 73% to 81%, respectively, and the relative bias decreased by up to 2 orders of magnitude. Least squares and maximum likelihood estimators had a relative bias of about ± 10^−4^.

**Table 3:** Performance of parameter estimation for the time‐concentration activity model

	**B(**$\delta_{Y_{i}}$**)**		**B(**$\alpha_{XY_{i}}$**)**		**B(**$\delta_{Y_{s}}$**)**		**B(**$\alpha_{XY_{s}}$**)**			
	**×10**^**−2**^								**Coverage probability**	
**Model*A(t,c)***	**LS**	**ML**	**LS**	**ML**	**LS**	**ML**	**LS**	**ML**	**LS**	**ML**
‘Different parameters’	.013	−.004	−.029	−.058	.009	.029	.005	.003	.86 [.84,.88]	.78 [.75,.80]
‘Identical intercept’	.012	−.014	.023	−.018	−.005	.018	−.001	.002	.87 [.85,.89]	.61 [.58,.64]
‘Identical slope’	−7.94	−7.91	23.9	23.5	.050	.094	−.057	−.055	.77 [.74,.79]	.73 [.70,.75]
‘Identical rates’	1.48	1.29	−1.64	−1.92	2.99	2.74	−2.75	−2.41	.81 [.79,.84]	.58 [.55,.61]

Bias **B** of least squares (LS) and maximum likelihood (ML) estimates and coverage probability with a 95% confidence interval of joint estimates for the time‐concentration activity model determined from 1000 simulations and fits of the scenarios each.

#### Testing hypotheses about transition rates

The scenario ‘Different parameters’, i.e. *δ*_*Y*__*i*_≠*α*_*XY*__*i*_, *δ*_*Y*__*s*_≠*α*_*XY*__*s*_, is the most general case for *A*(*t*,*c*). The scenarios ‘Identical intercept’ (*δ*_*Y*__*i*_≡*α*_*XY*__*i*_) and ‘Identical slope’ (*δ*_*Y*__*s*_≡*α*_*XY*__*s*_) are special cases of the scenario ‘Different parameters’. The scenario ‘Identical rates’ (*δ*_*Y*_(*c*)=*α*_*XY*_(*c*)) is a special case of all the other scenarios. LR tests were performed for 1000 simulated datasets to compare nested models. At the significance level of 5% the following results were obtained.

Simulating ‘Identical rates’ resulted in 62 ‘Identical intercept’, 57 ‘Identical slope’ and 80 ‘Different parameters’ cases that showed a significantly better fit than ‘Identical rates’. Hence the observed type I errors of the corresponding LR tests were 6.2%, 5.7% and 8%. Simulating ‘Identical intercept’ resulted in 74 cases in which ‘Different parameters’ showed a significantly better fit than ‘Identical intercept’. Hence the observed type I error of the LR test was 7.4%. Simulating ‘Identical slope’ resulted in 60 cases in which ‘Different parameters’ showed a significantly better fit than ‘Identical slope’. Hence the observed type I error of the LR test was 6%. All performed LR tests achieved a power of 100%.

## Application to experimental data

Data from the experiment illustrated in Figure 3 were time‐concentration activity data modeled by *A*(*t*,*c*). The initial number of cells was assumed to be *x*_0_=10^6^. Orders of magnitude of the maximum likelihood parameter estimates were too high, resulting in the death of all cells at 14 days for the control culture, i.e. P[X(t=14,c=0)+Y(t=14,c=0)=0]=1. Here the approximation of the log‐likelihood failed because the approximation of the distribution of *A*(*t*,*c*) relies on E[X(t,c)+Y(t,c)]≫0.

Least squares estimates were given by ${\hat{\delta_{Y}}}_{i}=1.1\times 10^{-11}$ in [day]^−1^, ${\hat{\alpha_{\text{XY}}}}_{i}=.0634$ in [day]^−1^, ${\hat{\delta_{Y}}}_{s}=.0371$ in [day ·*μ*M]^−1^ and ${\hat{\alpha_{\text{XY}}}}_{s}=.0008$ in [day ·*μ*M]^−1^, with residual sum of squares equal to 1.8683. The point estimate for $\delta_{Y_{i}}$ was on the boundary of the parameter space, hence the Fisher information matrix could not be used to calculate confidence intervals for the parameter estimates. Therefore, *A*(*t*,*c*) was also applied to the dataset with parameter restrictions and the restricted fits were compared to the full model fit. A restricted model fit with *δ*_*Y*__*i*_≡0 led to identical residual sum of squares and ${\hat{\alpha_{\text{XY}}}}_{i}=.0636\;\;[.0297,.1361]$ in [day]^−1^, ${\hat{\delta_{Y}}}_{s}=.0370\;\;[.0134,.1021]$ in [day ·*μ*M]^−1^ and ${\hat{\alpha_{\text{XY}}}}_{s}=.0008\;\;[.0004,.0017]$ in [day ·*μ*M]^−1^. A restricted model with *δ*_*i*_≡*δ*_*Y*__*i*_≡*α*_*XY*__*i*_ (identical intercept) yielded only minimally larger residual sum of squares equal to 1.8922 and $\hat{\delta_{i}}=.0787\;\;[.0332,.1864]$ in [day]^−1^, ${\hat{\delta_{Y}}}_{s}=.0477\;\;[.0193,.1181]$ in [day ·*μ*M]^−1^ and ${\hat{\alpha_{\text{XY}}}}_{s}=.0008\;\;[.0004,.0017]$ in [day ·*μ*M]^−1^. The restrictions *δ*_*Y*__*i*_≡*α*_*XY*__*i*_,*δ*_*Y*__*s*_≡*α*_*XY*__*s*_ (identical rates), *δ*_*Y*__*s*_≡*α*_*XY*__*s*_ (identical slope) and *δ*_*Y*__*s*_≡0 resulted in a significant increase in the residual sum of squares at the level of 5%.

**Figure 5:**
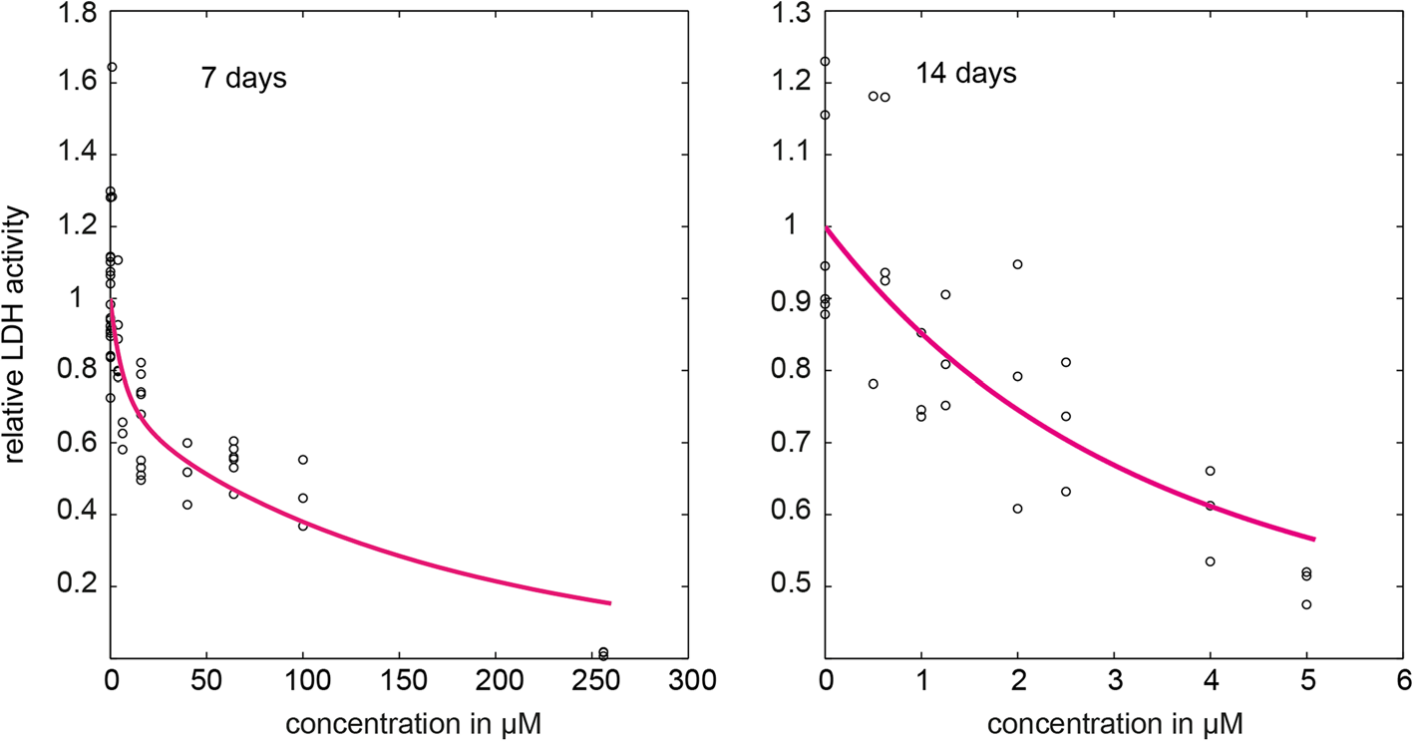
**Raw data and model fit.** Intracellular LDH activity data of cultures exposed to propofol and least squares curve fit of *A*(*t*,*c*). The thickness of the line covers the range between the 2.5th and 97.5th percentiles of 1000 simulations under the estimated parameters.

Figure 5 shows the raw data and the original least squares curve fit of *A*(*t*,*c*). The thickness of the line covers the range between the 2.5th and 97.5th percentiles of 1000 simulations, with the estimated parameters obtained from the full‐model fit to illustrate the amount of variability generated by the stochastic process. Hence the amount of variability caused by stochasticity in transitions between the subtypes of the cell populations was negligible compared to the experimental variability and, therefore, least squares regression is the appropriate method for parameter estimation rather than maximum likelihood estimation. Next we give an interpretation of the two restricted model fits *δ*_*Y*__*i*_≡0 and *δ*_*Y*__*i*_≡*α*_*XY*__*i*_. Due to the Markov property, the sojourn time of a cell in state one (healthy) is exponentially distributed with parameter *α*_*XY*_(*c*), the sojourn time of a cell in state two (stressed) is exponentially distributed with parameter *δ*_*Y*_(*c*) and the mean waiting times for transitions are given by *α*_*XY*_(*c*)^−1^ and *δ*_*Y*_(*c*)^−1^, respectively. Since the time‐concentration activity model is symmetric in the transition rates *α*_*XY*_(*c*) and *δ*_*Y*_(*c*), two interpretations of the estimates are possible.

For the restricted model *δ*_*Y*__*i*_≡0, one interpretation is that increasing the propofol concentration from 10 to 100 *μ*M reduces the mean waiting time for transition to the stressed state by 50% from 14 days (a 95% confidence interval: [6.5, 29.7] days) to 7 days (a 95% confidence interval: [3.3, 14.4] days), whereas the mean duration to cellular death decreases more dramatically from 2.7 days (a 95% confidence interval: [1.0, 7.5] days) to 6.5 hours (a 95% confidence interval: [2.4, 17.9] hours). Therefore, propofol affects stressed cells more than it affects healthy cells. In the second interpretation, the roles of *δ*_*Y*_(*c*) and *α*_*XY*_(*c*) are interchanged: increasing the propofol concentration from 10 to 100 *μ*M would reduce the mean waiting time for transition to the stressed state from 2.7 days to 6.5 hours, whereas the mean duration to cellular death would reduce from 14 to 7 days.

For the restricted model *δ*_*Y*__*i*_≡*α*_*XY*__*i*_, the interpretations are analogous: one interpretation is that increasing the propofol concentration from 10 to 100 *μ*M reduces the mean waiting time for transition to the stressed state from 11.5 days (a 95% confidence interval: [4.9, 26.9] days) to 6.3 days (a 95% confidence interval: [2.8, 13.7] days), whereas the mean duration to cellular death decreases from 1.8 days (a 95% confidence interval: [0.7, 4.4] days) to 5 hours (a 95% confidence interval: [2.0, 12.2] hours). Hence, propofol affects stressed cells more than it affects healthy cells. In the second interpretation, the roles of *δ*_*Y*_(*c*) and *α*_*XY*_(*c*) are interchanged: increasing the propofol concentration from 10 to 100 *μ*M would reduce the mean waiting time for transition to the stressed state from 1.8 days to 5 hours, whereas the mean duration to cellular death would decrease from 11.5 to 6.3 days.

From a biological point of view, it appears plausible that cells may die due to different reasons. Exchange of the medium could cause death of untreated cells because the experimental conditions cannot be kept exactly constant. In addition, cells from the control culture could die due to aging processes. Hence, the restricted model *δ*_*Y*__*i*_≡*α*_*XY*__*i*_ appears to be more plausible biologically than the restricted model *δ*_*Y*__*i*_≡0. Xenobiotics such as propofol challenge the homeostasis of the cells, which in turn triggers cellular responses enabling the cells to adapt and survive. The cellular mechanisms of defense include antioxidative and anti‐inflammatory responses. Once the adaptive mechanisms are overcome by the exposure to xenobiotics, cellular death ensues. Therefore, the first interpretation of the estimates for both restricted models appears to be more plausible, since adaptive mechanisms may take longer than cell death per se. However, the level of cellular stress must be monitored in further experiments in order to validate or invalidate the second interpretation.

## Discussion

A Markov process which modeled the survival of brain cells to a chemical insult was developed. We derived the distribution of cell numbers analytically with transition rates as functions of concentration but constant in time. Assuming that changes in cell numbers are proportional to changes in intracellular LDH activities which were measured as a surrogate, stochastic activity models accounting for two different normalization procedures of activity data were derived. Activity data of untreated cultures were normalized with respect to activities at time zero, whereas activity data of treated cultures were normalized with respect to untreated cultures at the same time point. The transition rates of the Markov process were assumed to be constant for time activity data and as linear functions of concentration for time‐concentration activity data. Simulation studies were carried out to assess the performance of parameter estimation. LR tests were performed to test hypotheses about the transition rates at a significance level of 5%. The observed type I errors were between 1.1% ‐ 8% and power was equal to 100%. For the time activity model, maximum likelihood estimators performed better than least squares estimators in terms of coverage probability and bias. For the time‐concentration activity model, both estimators performed similarly in terms of coverage probability and bias. We applied the time‐concentration activity model to an experiment in which brain aggregate cell cultures had been exposed to propofol for up to 14 days to assess its *in vitro* CNS toxicity. Intracellular LDH activity had been measured at two time points and allowed for a conclusion about average transition times between 6.5 hours and 14 days for propofol concentrations between 10 and 100 *μ*M.

The primary model we derived describes the cell populations, consisting of healthy and stressed cells, and the transitions between these subtypes and cell death. The actual counts of cells in these two different states are not observable but their sum is reflected by LDH activity measurements. As LDH activity measurements are highly variable, they were normalized either to measurement at the start of the experiment (for the time activity model) or to the untreated control at the same time point (for the time‐concentration activity model). Although the two activity models deliver information only about the total number of cells in the system, conclusions about the original cell kinetic model can be drawn, with the limitation that not all transition rates can be identified. However, the effect of the compound on the transition rates can be estimated.

The models presented here describe the fate of a brain cell population. Brain aggregates consist of several types of brain cell populations. In the present approach, no distinction between the different cell types can be made on the basis of LDH activity measurements. However, enzymes are known that are brain cell‐type specific. Future research will be needed to extend the present activity models to incorporate cell type‐specific enzyme activity data. Enzyme activities are measured in every flask containing hundreds of brain aggregates. Due to the necessary mathematical assumption of independence of the cells, all conclusions drawn from the modeling approach do not depend on the actual number of brain aggregates and their size, but only on the total number of cells in the flask.

The time‐concentration activity model was applied to experimental LDH activity measurements during exposure to propofol. Least squares estimates of the transition rates were obtained, which in contrast to maximum likelihood estimates do not account for systematic variation in the measurements. We observed that the proposed model variability differed considerably from experimental variability. This indicates that either the model should account for further sources of variability or that variability is caused by random independent measurement errors. For example the model could account for differences in the initial number of cells *x*_0_ between different samples. However, when we changed the value of *x*_0_ by a factor of ten, the model still predicted much less variability in comparison to the observed variability. Hence, we believe that measurement error plays the major role in the variability of the data and least squares parameter estimation is the appropriate method to reflect this finding. Since stochasticity, due to transitions between states, contributes little to the overall variability, it might be more productive in the long run to use a deterministic approximation to the cell population model as the basis for a nonlinear mixed effects model. This could incorporate variations in initial cell number, measurement error in LDH activity and differences in physical conditions between replicates.

For the time‐concentration activity model we assumed transition rates as linear functions of concentration. This assumption was our first approach to incorporating concentration into the model. It assumes that exposure to the compound under study starts or augments an ongoing process of transition to the stressed state and eventual cell death. A more general approach is to use transformations of concentration instead of the concentration itself, such as log(*c*+1), exp(*c*) −1, $\sqrt{c}$ or *c*/(1+*c*). In principle, any biologically plausible dose‐response relationship for the parameter can be easily implemented as long as no additional unknown parameters are introduced and as long as transition rates remain independent of the time course. Using the transformations of concentration given above, we applied the time‐concentration activity model to the intracellular LDH activity measurements during exposure to propofol. All transformations have in common that maximum likelihood estimation failed due to death of all control culture cells at day 14, i.e. P[X(t=14,c=0)+Y(t=14,c=0)=0]=1. In addition, one intercept parameter was estimated to be zero for each transformation and the residual sum of squares was between 2.0146 ‐ 8.6941. Generally, cell kinetic models are very sensitive to alterations in the model parameters (see, e.g. Kopp‐Schneider [13]). The exponential transformation has too great an impact on the transition rates, whereas the remaining transformations lack impact on the transition rates when high concentrations of propofol, such as 256 *μ*M, are investigated. In summary, *A*(*t*,*c*) with transition rates as linear functions of concentration explains the propofol data best.

The proposed stochastic modeling approach can be used to discriminate between different biological hypotheses about the effect of a compound on the transition rates. If there is biological evidence for a subset of parameters to be identical or to be zero, then both the full and the restricted nested model can be applied to the data. The two model fits can be compared by making an extra sum of squares analysis to determine whether there is a statistically significant difference between the error sum of squares in order to accept or reject a simplified hypothesis.

The time‐concentration activity model can be applied to data from experiments performed to investigate the neurotoxicity of compounds. The effects of each compound on the transition rates of the Markov process can thus be estimated. By comparing the impact on the transition rates, the neurotoxic effects of the compounds can be compared in a quantitative and mechanistic manner.

In future experimental research, propofol experiments will be redesigned and performed with more measurement time points up to day 14. We will apply our models to data from early measurement time points and extrapolate to late measurement time points. The extrapolated and experimental outcome at late time points will be compared in order to to assess whether our models are able to predict measurements correctly. If the extrapolation corresponds adequately to the experimental data, then costs and time‐consuming long‐term experiments could possibly be eliminated.

## Abbreviations

CNS: Central Nervous System; LDH: Lactate Dehydrogenase

## Competing interests

The authors declare that they have no competing interests.

## Authors’ contributions

Conceived, designed and performed the experiments: MZ. Developed the mathematical models: MR AK. Wrote computer simulation and analysis programs: MR. Analyzed the data: MR. Wrote the paper: MR MZ AK. All authors read and approved the final manuscript.
